# Strengthening post‐exposure prophylaxis uptake among survivors of sexual violence through immediate access at police stations in Nigeria's Federal Capital Territory

**DOI:** 10.1002/jia2.26460

**Published:** 2025-06-26

**Authors:** Bukola Adewumi, Meagan Cain, Udhayashankar Kanagasabai, Sushma Dahal, Derby Collins‐Kalu, Abiola Mutka Ayuba, Victor Adamu, Timothy Efuntoye, Christabel Ayeni, Helen Omuh, Chigozie Nwafor, Adebola Raji Ajuwon, Orisawayi Oluwaniyi, Patrick Dakum, Rita Oki‐Emesim, Fatima Daggash, Omodele Fagbamigbe

**Affiliations:** ^1^ Division of Global HIV & Tuberculosis U.S. Centers for Disease Control and Prevention Abuja Nigeria; ^2^ Division of Global HIV & Tuberculosis U.S. Centers for Disease Control and Prevention Atlanta Georgia USA; ^3^ Institute of Human Virology Abuja Nigeria; ^4^ Nigerian Police Force Headquarters Abuja Nigeria; ^5^ Public Health Department FCT AIDS and STI Control Program Abuja Nigeria

**Keywords:** HIV, police stations, post‐exposure prophylaxis, prevention, Africa, sexual violence

## Abstract

**Introduction:**

Data on sexual violence (SV) prevalence in Nigeria is limited; however, 2014 data indicate that 24.8% of females aged 18−24 years experienced SV in childhood and only 3.5% received any form of services. Initiation of post‐exposure prophylaxis (PEP) to prevent HIV acquisition following SV is most effective when started immediately and is not recommended after 72 hours. Police stations are often entry points for survivors; however, lengthy processes may result in delays and missed PEP opportunities. Using an ongoing phased approach, we introduced PEP into selected police stations in Nigeria's Federal Capital Territory in order to explore expanding access to time‐sensitive HIV prevention within non‐health services.

**Methods:**

Our intervention phase consisted of the provision of training of police officers and the provision of PEP starter packs coupled with linkage to referral facilities. During two time periods (pre‐intervention: January−March 2023) and (during intervention: July−September 2023), we evaluated routinely reported programme data from 27 U.S. Centers for Disease Control and Prevention‐supported health facilities for changes in the provision of SV services and PEP initiation. We used geospatial mapping to assess the proximity of participating health facilities to police stations and to see changes in both SV and PEP service provision. The statistical significance of the difference in PEP uptake proportion during the two periods was determined using the Wilcoxon signed rank test at a 0.05 level of significance.

**Results:**

Of the total 27 health facilities, 24 were within a 5‐km radius of a participating police station. Total SV service provision increased from 114 cases to 218 cases, representing a 91.2% increase and with most of this increase seen among females. PEP initiation increased by 289.3% at the two time points, with 56 initiations pre‐intervention to 218 PEP initiations during the intervention.

**Conclusions:**

Our findings showed promise in increasing immediate access to PEP in non‐health services and highlighted the feasibility of police stations and health facilities collaboration to address urgent health needs. There was an overall increase in PEP initiations by referral and non‐referral facilities which could be the result of demand creation and increased access at police stations.

## 1 INTRODUCTION

Sexual violence (SV) remains a serious public health concern worldwide, and especially in Africa [1], with individuals facing immediate and long‐term health impacts including psychological disorders, unplanned pregnancy, chronic health conditions such as asthma, and HIV [2, 3]. Globally, approximately one‐third (27%) of women will experience sexual and/or physical violence; and while most of this comes from an intimate partner, 6% of women will also experience non‐partner SV at least once in their lives [4]. According to the 2014 Nigeria Violence Against Children Survey, one in four females reported experiencing SV during their childhood, with about 70% reporting multiple incidents of SV [5]. The survey findings also revealed that of the 24.8% of females aged 18−24 years who experienced SV in childhood, only 3.5% received any services [5, 6].

With approximately two million people living with HIV, Nigeria has one of the largest HIV epidemics globally [7]. Post‐exposure prophylaxis (PEP) is an effective HIV prevention intervention when taken within 72 hours of a potential exposure to HIV, such as following an experience of SV [,^8^, ^9^]. However, data from Nigeria show inadequate levels of PEP uptake among survivors of SV, with adolescent survivors having especially low rates [10]. Many survivors initially seek help from non‐health services such as police stations, leading to delays in initiating time‐sensitive health interventions. A study conducted in Nigeria indicated that late reporting of rape cases at non‐health facilities, such as police stations or community centers, is the major reason for the low initiation of PEP within the required 72‐hour effectiveness window [11]. Other reasons for poor PEP use include misconceptions around PEP, stigma, societal judgement and the emotional state of survivors following rape, which can further traumatize the survivor and complicate timely PEP seeking and initiation [11, 12, 13]. Additionally, a cross‐sectional study among Nigerian university students found that only a quarter of participants were aware of PEP, only 10% knew where to go to obtain PEP and less than 4% knew how much PEP costs [14].

Police play important roles in pursuing justice, protection and support for SV victims. However, the lack of survivor‐friendly police stations combined with stigmatization, rape myths, victim blaming, unprofessional conduct by police and confusing legal processes all present barriers to police service utilization [15, 16, 17]. In Nigeria, before a perpetrator can be successfully prosecuted in court, the police are required to conduct thorough investigations into the incident and provide a detailed report [16, 18]. The strong focus on justice and the lengthy legal processes may result in a deprioritization of critical health interventions and delays in timely referrals to health facilities where PEP can be initiated. Integrating PEP services at victim response desks within police stations and sensitizing police units to provide a PEP starter dose may be an effective intervention to address some of these challenges. Recognizing police stations as potential sites for intervention, we explored how immediate access to PEP at police stations can enhance timely uptake among survivors of SV in Nigeria's Federal Capital Territory (FCT). Utilizing geospatial mapping, the study assesses the proximity of health facilities to police stations and changes in service receipt to evaluate the impact of this intervention.

## 2 METHODS

We employed a cross‐sectional design using routinely collected health service delivery data from two time points to examine changes in SV service delivery prior to and during our intervention activities. More specifically, we were interested if we would see changes in the proportion of individuals receiving PEP at participating health facilities if PEP starter packs were made available at police stations.

The target group was survivors of SV (males and females of all ages), who presented to participating police stations. Inclusion criteria to receive PEP included individuals who: were sexually assaulted with penetration, presented to one of the participating police stations within 72 hours of the incident, were willing to receive medical assistance, including PEP, and provided informed consent (or assent with guardian consent, where applicable, for minors).

Survivors who presented after 72 hours post‐incident, declined PEP after counselling and were already HIV positive were excluded but were still linked to health facilities for other health services.

### 2.1 Intervention

Police stations within Nigeria's FCT were selected as intervention sites based on the following criteria:

1. Out of six districts in the FCT, three districts with the highest prevalence of violence were selected,
2. High volume (>5 survivor cases in a month) of reported post‐violence cases,
3. Police station location was within a 5‐km radius to a supported health facility, and
4. Availability of clinician to administer PEP.

Each police station in Nigeria has a dedicated Response Desk staffed by a specialized team that handles cases of violence. This team often includes a clinician responsible for providing medical care, conducting HIV tests, administering necessary medications and initiating survivors on HIV antiretroviral therapy (ART). Before the intervention began, a total of 29 police sites met the criteria and participated in pre‐intervention activities, including training and orientation to materials. A total of 29 police officers, one from each division, received a 5‐day classroom training on key topics related to emergency response to SV, HIV prevention, the use of HIV test kits, the regimen for PEP, data reporting and referrals. The training was conducted by the local implementing partner overseeing HIV and violence prevention activities in the state. Following the training, additional activities were implemented, including the development of standard operating procedures (SOP) and reporting/documentation tools. Antiretroviral drugs were supplied to the police stations from the participating referral health facility.

The participating referral facilities were hospitals within the same districts as the police stations in the FCT that provided comprehensive HIV treatment, care and support to individuals of all ages. All 27 health facilities were supported by the U.S. President's Emergency Plan for AIDS Relief (PEPFAR) through the U.S. Centers for Disease Control and Prevention (CDC). As part of regular supported programming, these facilities provided a package of post‐violence care services, which included HIV testing and PEP. The provision of these services was routinely reported through a PEPFAR data reporting system, disaggregated by violence service type (sexual or physical and/or emotional) by age and sex, and PEP initiation. In alignment with the latest World Health Organization guidelines for PEP [19], receipt and completion of PEP is defined as individuals who initiated PEP within 72 hours of exposure and completed the full 28‐day regimen.

Starting in July 2023, the participating police sites provided starter packs of PEP to individuals who presented within 72 hours of sexual assault. Upon reporting an incident, individuals provided written consent prior to undergoing HIV testing, which was conducted by a clinician at the response unit. Those who tested negative received an initial five‐pill PEP starter pack at the police station to ensure timely initiation of treatment. Following the SOP, the police then referred the survivor to the designated nearest referral facility for the remainder of the 28‐day PEP regimen and additional SV care services. The documentation at the police station was transferred to the referral facilities for immediate follow‐up with the survivor. Regimen completion was documented at the health facility, and confirmation was done through a phone call to the survivor at the end of 28 days. This data was then reported through the regular programme data reporting via the data system in which PEP data is reported semi‐annually.

Since PEP initiation at police stations was not part of routine care in Nigeria at the time, the intervention required additional training, protocol development and collaboration with health facilities to ensure proper administration, documentation and follow‐up. The primary goal was to integrate an emergency response model into the existing system, ensuring that survivors received care in alignment with national guidelines while strengthening referral pathways. Ethical considerations were upheld by adhering to institutional frameworks and ensuring that all services, including HIV testing and PEP provision, followed standard protocols for voluntary participation and confidentiality.

### 2.2 Data collection

We collected the data on the geo‐coordinates of participating police stations (intervention sites) that had begun providing PEP for immediate initiation. We then conducted geospatial mapping to show the health facilities within a 5‐km radius of the intervention facilities. We also mapped the proportion of PEP use out of total SV service provision for each of the health facilities providing post‐SV care before intervention and during intervention in separate maps.

Routinely collected programme data on SV service provision came from participating health facilities. Each SV case represents an individual who received at least one service from a basic package of services delivered at the health facility; these services include HIV testing, counselling, injury treatment, sexually transmitted infection testing and/or treatment, emergency contraceptives and PEP (either initiation or provision of remainder of pack). Regardless of if an individual presented at a police station, they were only counted as an SV case if they received at least one SV service at a participating health facility.

### 2.3 Data analysis

Utilizing routinely collected programme MER data from the participating referral health facilities, we analysed changes in SV service provision and PEP completion from two time points: pre‐intervention and during implementation of the intervention. Given the phased approach, we allowed sufficient time for all sites to be implementing in line with data reporting schedules, which resulted in a 3‐month gap (March−June) between pre‐implementation and full implementation for data analysis. From March to June 2023, training of sites and preparation of intervention sites occurred. We compared the data before and after initiation of the intervention (January−March 2023 vs. July−September 2023). For each health facility, we calculated the number of police stations within its 5‐km radius. We then conducted geospatial mapping to show the health facilities within a 5‐km radius of the intervention facilities (i.e. police stations). We also mapped the proportion of PEP use out of total SV service provision for each of the health facilities providing post‐SV care before intervention and during intervention in separate maps.

The proportion of PEP uptake was calculated by dividing the number of individuals receiving PEP by the total number of individuals receiving SV services at the health facility. We analysed changes in the proportion of PEP uptake in the health facilities during the study periods, and assessed differences using the Wilcoxon signed‐rank test at an alpha of 0.05. We used the non‐parametric alternative because of the small sample size. For each health facility, we also calculated the number of police stations within its 5‐km radius. We then assessed the association of difference in PEP proportion per health facility and the number of police stations that included that health facility within its 5‐km radius using Spearman rank correlation. We also assessed the association of the number of police stations with the difference in the number of PEP use reported in the health facility before and during the intervention and also the difference in SV cases reported in the health facility before and during the intervention using Spearman rank correlation.

All the analyses were performed using SAS version 9.4 software (SAS Institute, Cary, North Carolina). Geospatial mapping was done using QGIS Desktop 3.22.5.

### 2.4 Ethical review

The data are covered by a protocol reviewed by CDC, deemed non‐research, and conducted consistent with applicable federal law and CDC policy (45 C.F.R. part 46.102(l)(2), 21 C.F.R. part 56; 42 U.S.C. Sect. 241(d); 5 U.S.C. Sect. 552a; 44 U.S.C. Sect. 3501 et seq). Written consent was obtained from all health and non‐health facilities used in the study.

## 3 RESULTS

There was a 289.3% increase in the number of PEP initiations, from 56 individuals to 218 individuals, from Q2 (before the intervention period: January−March 2023) to Q4 (during the intervention period: July−September 2023). Within the same time frames, the number of reported SV cases increased from 114 to 218 representing a 91.2% increase. The proportion of individuals initiating PEP in relation to the number of SV cases increased from 49.1% pre‐intervention to 100% during intervention, indicating an increase of around 104% (Table 1).

**Table 1:** Summary of results from health facilities, pre‐ and post‐intervention (*n* = 27), Nigeria, Abuja, 2023

Year 2023	Pre‐intervention	During intervention	% Increase
No. of health facilities with reported SV cases	19	21	10.5%
Number of SV cases	114	218	91.2%
Number initiated on PEP	56	218	289.3%
Proportion of PEP to SV cases	49.1% (56/114)	100% (218/218)	103.6%

Of the participating 27 health facilities, 19 facilities reported cases of SV in the pre‐intervention time point. In the intervention phase, the number of facilities reporting SV increased to 21. This showed a 10.5% increase in the number of facilities reporting SV after the intervention was initiated. During the pre‐intervention period, out of 19 health facilities that reported at least one SV case, four (21.1%) facilities had a PEP uptake proportion of 0% out of total SV cases and nine (47.37%) had a PEP uptake proportion of 100%. During the intervention period, the PEP uptake proportion out of total SV cases increased to 100% across all 21 facilities reporting at least one SV during the intervention period. It is important to note that one of the health facilities with reported SV in the pre‐intervention phase did not have any SV reported during the intervention phase; therefore, a total of 18 facilities had reported SV for both time points.

**Figure 1:**
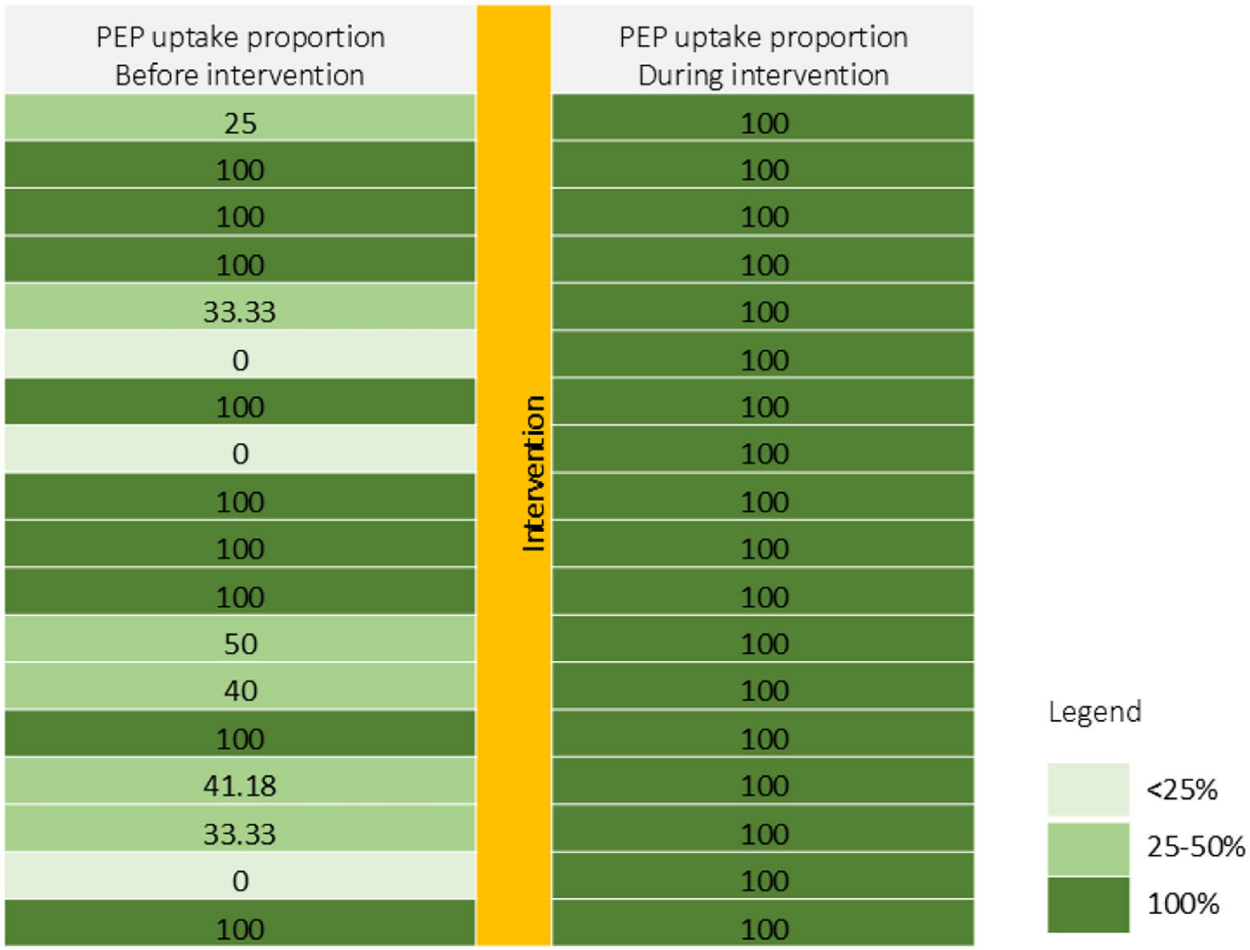
Heat map showing the proportion of PEP uptake before and during the intervention for 18 health facilities. Each row indicates a health facility.

Figure 1 shows the PEP uptake proportion in these 18 health facilities during the two study periods in a heat map. For these 18 health facilities, we found that the median difference in the PEP uptake proportion during the intervention period and before intervention was 25% (interquartile range: 0%, 66.67%) and this difference was statistically significant (*p*‐value = 0.004). Figure 1 shows the PEP uptake proportion in the 19 health facilities with reported SV during the pre‐intervention period.

Females were the majority of individuals receiving SV services during both pre‐ and active intervention points, at 87.7% and 87.6% of total SV cases. Pre‐intervention PEP proportion initiation ranged from 0% among <15 males (*n* = 3 SV cases) to a high of 73% among ≥15 males (*n* = 11 SV cases). SV cases among ≥15 females saw the largest increase between the two time points, increasing from 48 cases to 149 cases during the intervention (Table 2).

**Table 2:** Summary of results by coarse age and sex, pre‐ and post‐intervention, Nigeria, Abuja, 2023

	Pre‐intervention				During intervention			
	Female		Male		Female		Male	
	<15	≥15	<15	≥15	<15	≥15	<15	≥15
SV cases	52	48	3	11	42	149	4	23
SV cases initiated on PEP	21	27	0	8	42	149	4	23
Proportion of PEP to total SV	40%	56%	0%	73%	100%	100%	100%	100%

**Figure 2:**
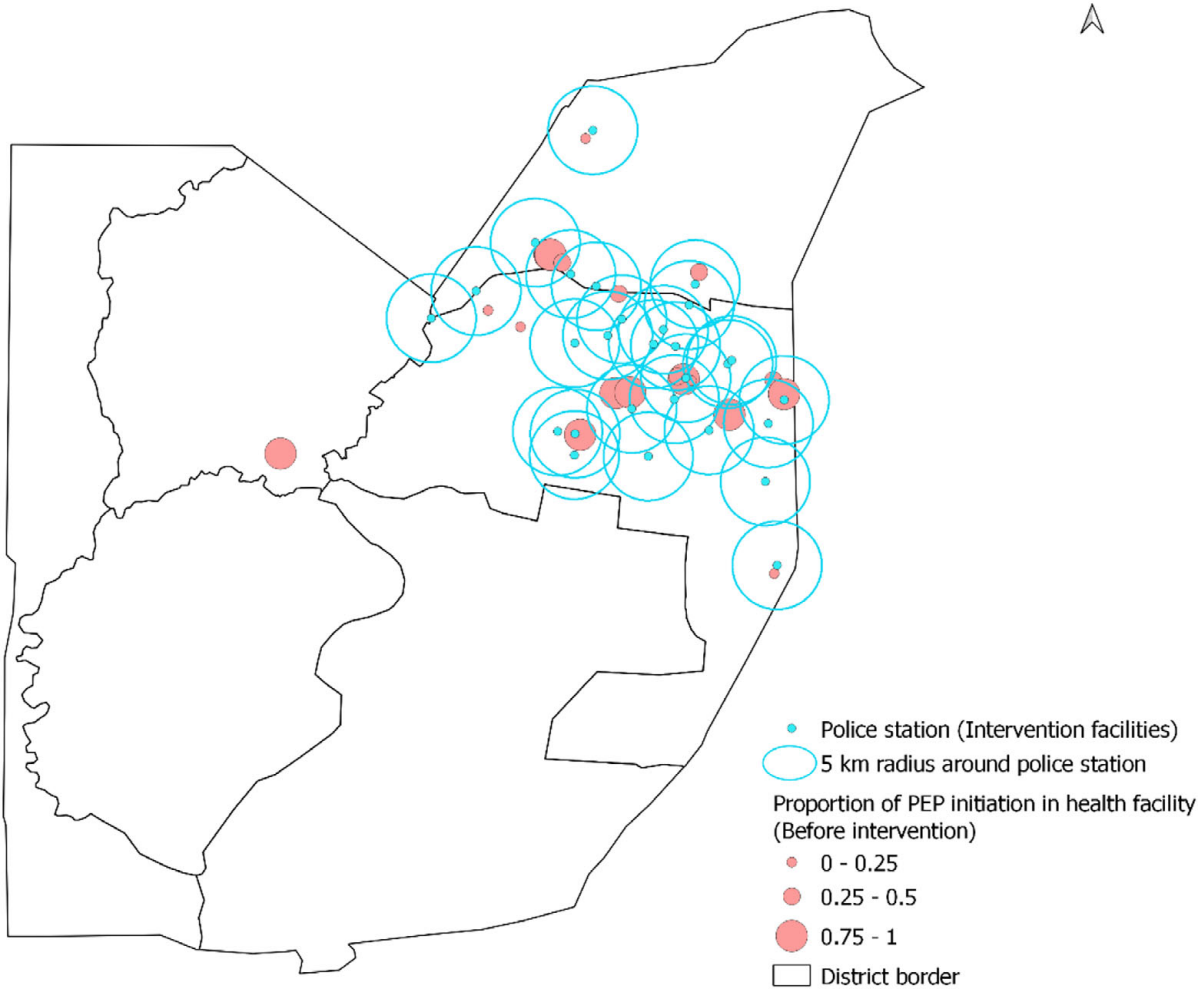
Map of Federal Capital Territory showing locations of police stations and PEP uptake proportion reported in the health facilities at the pre‐intervention phase, 2023.

**Figure 3:**
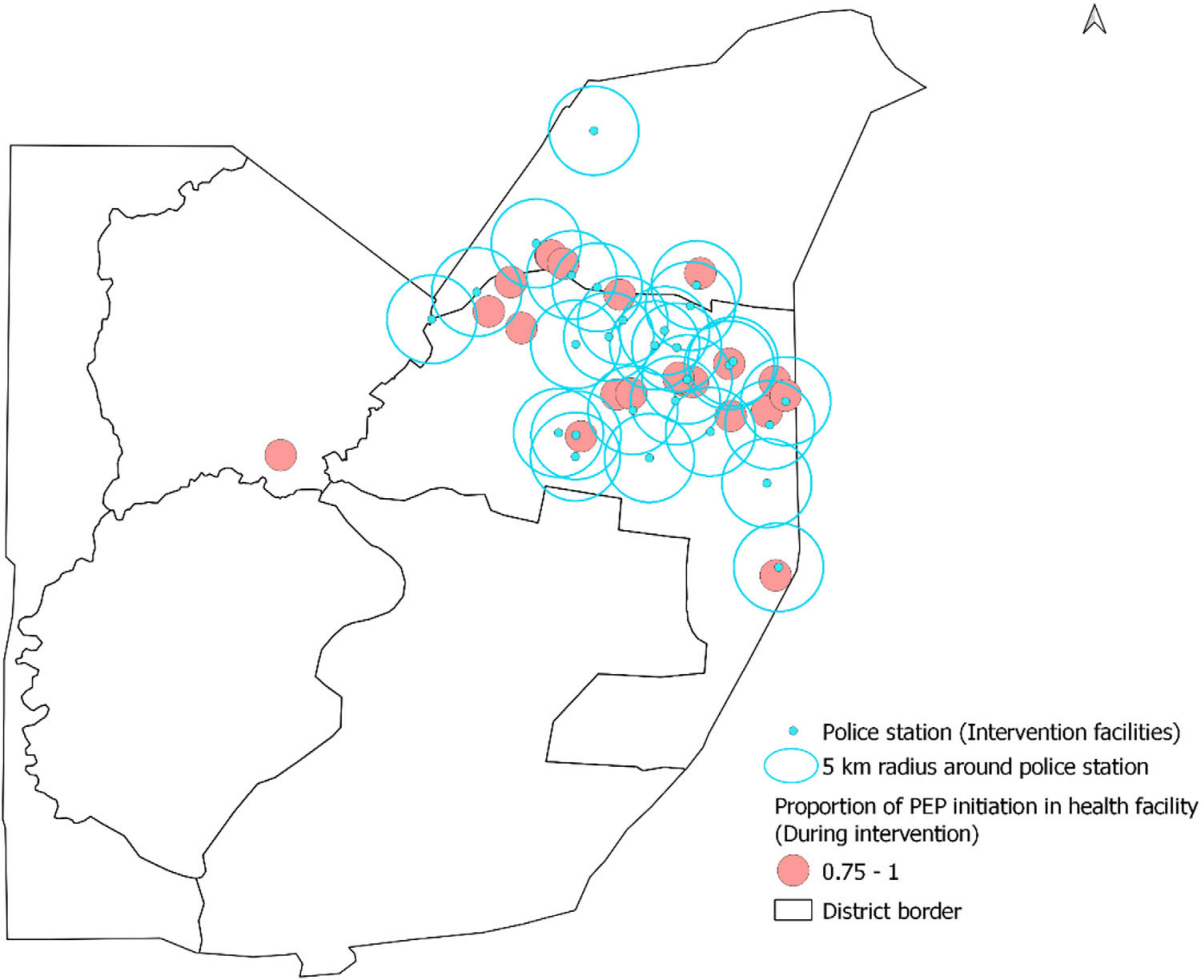
Map of FCT showing locations of police stations and PEP uptake proportion reported in the health facilities during the intervention phase.

Figures 2 and 3 show the location of the 29 police stations, the 5‐km radius around them and the PEP proportion in the referral health facilities overlayed in the map of FCT for the two study periods, respectively. The proportion of PEP uptake was higher in the intervention phase compared to the pre‐intervention phase.

Across 27 health facilities within 5 km of a police station, the average number of corresponding police stations within the 5‐km radius was two. Three facilities (11.1%) were not within a 5‐km radius of any police station, 16 (59.3%) were within a 5‐km radius of one to two police stations and eight (29.6%) were within the 5‐km radius of three to five police stations. Our estimate of the Spearman rank correlation coefficient showed a very weak association between the difference in PEP uptake proportion and the number of police stations that covered the health facility within its 5‐km radius (*n* = 18, rho = 0.02, 95% CI: −0.45, 0.48, *p*‐value = 0.94). Likewise, we did not find a significant association between the difference in the number of SV cases reported before and during the intervention and the number of police stations (*n* = 27, rho = 0.30, 95% CI: −0.09, 0.61, *p*‐value = 0.125). However, we found a significantly positive association between the difference in the number of PEP use before and during the intervention and the number of police stations that covered the health facility within its 5‐km radius (*n* = 21, rho = 0.52, 95% CI: 0.10, 0.77, *p*‐value = 0.015).

## 4 DISCUSSION

The intervention showed a significant increase in PEP uptake among survivors of SV who received services from health facilities. Between the two study periods, there was a 91.2% increase in the reported number of SV services, a 289.3% increase in PEP service initiation and a 103.6% increase in the proportion of PEP uptake out of total SV reported. PEP uptake as a proportion of SV increased from 49.1% in the pre‐intervention phase to 100% in the intervention phase. The findings showed that not only did PEP initiation increase but the overall number of SV services delivered also increased, especially for adult women. Similar to other findings, the majority of SV cases were female—further evidencing the disproportionate impact of SV on women and girls in Nigeria [16, 20]. While we did not measure individual changes in attitudes nor actual police behaviours with victims, previous studies have shown that training on SV can improve police interactions and service uptake to meet both the legal and health needs of survivors [21, 22].

The study highlighted the importance of providing immediate PEP access at locations where survivors first present for services, which may not be at health facilities. Police stations represent important service points for survivors and may hold promise in reducing timely access to critical HIV prevention interventions following an SV exposure. Integrating PEP provision into services offered at police stations can improve the overall response to SV and enhance health outcomes for survivors by giving survivors more time to present to health facilities and potentially through increasing demand for health services. The findings of this study are supported by a study carried out in Zambia in 2015 to assess the feasibility of providing PEP at police stations [23[. Their findings also pointed to the need to have police officers provide a PEP starter dose to survivors of SV with immediate referral to health facilities.

Beyond increasing PEP initiation rates, the intervention has the potential to improve awareness about PEP and the importance of timely SV service‐seeking through police interactions with individuals and communities. In addition to availability and access, increasing knowledge of both PEP and where to access PEP are critical for increasing PEP use [24,25]. Conducting awareness campaigns to inform communities about PEP availability at police stations and other non‐health service points may be beneficial in addition to activities such as training of police officers on administering PEP and caring for survivors of SV, and establishing clear policies and procedures to guide the integration of PEP provision within police stations.

This study has several strengths. One of the primary strengths of this study is its innovative approach to integrating health services within law enforcement settings, in which health services may not be the primary concern of the police. Second, the diversity of the study sites, including both urban and rural police stations, adds to the generalizability of the findings. Previous research has shown that a full regimen of PEP has more positive effects on PEP completion over starter packs in health settings [26]. However, given the non‐clinical setting of police stations, the need for additional health interventions and the overall design of the intervention, starter packs were chosen as the best available option. Our integrated approach did not aim to replace the sensitive care and health interventions that are best delivered by trained health professionals; instead we strengthened links from police to existing health settings while not sacrificing critical time for PEP initiation. We believe this holistic approach can better meet the needs of survivors where they first come into contact with formal help services—which can ultimately lead to long‐term benefits for community trust and engagement while acknowledging the inherent necessity, and also sometimes friction, that exists between the police and SV victim supports [27].

This study has some important limitations. First, we only included data from CDC‐supported health facilities; meaning that individuals could have sought care at other service sites. Additionally, the sample size of the study was very small, limiting the analysis’ ability to explore relationships between other factors. While police stations across Nigeria have embedded victim units, the availability of a clinician to prescribe PEP and the quality may vary, especially outside of the FCT. Routinely collected programme data were limited to data from health‐facility reporting in that we were not able to disaggregate SV cases who first reported to the police and linked to the health facility versus those who first presented at the health facility, which gives us a limited view of how many were referred through the police stations. A natural limitation of service delivery data is that it only reflects individuals who received services and not the total of those in need of services; this means it is possible that there could be a self‐selection bias of who independently seeks services at both facilities and the police stations. Globally and in Nigeria, the majority of individuals who receive SV services are women and girls [1, 5, 6, 8] as SV is often underreported and it is possible that other populations such as men and boys would be deterred from seeking services through the police. Finally, without a comparison group, we cannot truly attribute changes to service receipt to the intervention. Despite the limitations, we feel this approach adds important nuance to the call to meet survivors with services where they first present—which includes law enforcement sites.

## 5 CONCLUSIONS

The study highlights a promising strategy for increasing PEP uptake among survivors of SV. The initiation of PEP within police stations reduces the barriers to PEP access that survivors encounter following a sexual assault. By reducing barriers to PEP access and fostering a supportive environment for survivors, this intervention has the potential to significantly improve health outcomes and prevent HIV transmission. Further research may help define the long‐term sustainability of the intervention and to refine the model for broader implementation. Partnering with police can be considered as an option to improving survivors of SV timely access to PEP and may contribute to ending HIV as a public health threat in Nigeria.

## COMPETING INTERESTS

The authors have no conflicts of interest to declare.

## AUTHORS’ CONTRIBUTIONS

BA and DC‐K developed the study's concept design. The writing of the manuscript, data analysis and data interpretation were done by BA, MC, UK and SD. All authors participated in reviewing and revising the manuscript. After the manuscript's review, all authors approved the final version.

## FUNDING

This work was supported by the United States President's Emergency Plan for AIDS Relief (PEPFAR) through the Centers for Disease Control and Prevention.

## DISCLAIMER

This study manuscript is provided for informational and research purposes. The findings and conclusions in this manuscript are those of the author(s) and do not necessarily represent the official position of the funding agencies.

## Data Availability

Data are available upon request from authors.
